# Characteristics of 340B Hospitals Receiving Medicare Part B Repayments

**DOI:** 10.1001/jamahealthforum.2023.5397

**Published:** 2024-04-26

**Authors:** Sayeh Nikpay

**Affiliations:** 1 Division of Health Policy and Management, University of Minnesota School of Public Health, Minneapolis

## Abstract

This cross-sectional study compared the characteristics of 340B hospitals that did not receive a lump sum payment with hospitals in the program that did receive payment.

## Introduction

The 340B Drug Pricing Program entitles certain hospitals and federally supported safety-net clinics to discounts on drugs in outpatient settings. In addition to lowering drug costs, participants can bill patients with health insurance at customary rates, generating savings designed to fund safety-net care. However, these savings—as much as $50 billion annually^1^—are unreported and not shown to improve care access for patients without adequate insurance.^2,3^

In Medicare Part B, 20% of the drug reimbursement 340B hospitals receive comes from beneficiaries with no out-of-pocket maximum. To lower drug costs for Medicare beneficiaries,^4^ Centers for Medicare & Medicaid Services (CMS) implemented 340B-specific cuts to Part B drug reimbursements in 2018 (average sales price [ASP] + 6% to ASP − 22.5%). The cuts reduced total Medicare spending by roughly $1.6 billion annually for nearly 5 years, until reversal by the US Supreme Court in 2022. In addition to restoring reimbursements to ASP + 6%, CMS will repay 340B hospitals forgone reimbursement in lump sum payments totaling $9 billion in 2024.^5^

Per CMS’s budget neutrality principle in the Outpatient Prospective Payment System (OPPS), the $9 billion repayment must be offset by $7.8 billion of cuts to nondrug reimbursements. Therefore, hospitals administering large quantities of high-cost drugs will benefit, while hospitals that do not could face a net loss of Medicare reimbursement, which raises equity concerns.^6^

## Methods

CMS lump sum payment estimates were linked to Hospital Cost Reports and HRSA Office of Pharmacy Affairs data to identify OPPS hospitals participating in the 340B program for at least 1 year between 2018 and 2022. To evaluate the importance of cuts to nondrug reimbursements, each hospital's nondrug Part B reimbursement was calculated by adjusting lump sum reimbursements to represent the payments hospitals would have received and deducting them from total Part B reimbursements over the same period. This research used publicly available administrative data and did not require institutional review or informed consent.

This analysis compared the characteristics of OPPS 340B hospitals not receiving a lump sum payment with hospitals receiving payment. Characteristics evaluated included net operating revenue, uncompensated care burden (charity care and bad debt costs as a share of net operating revenue), total profit margin, rurality, public ownership, and teaching status. Among hospitals receiving payment, the linear associations between payments and (1) uncompensated care burden, (2) total profit margins, and (3) the nondrug share of Part B reimbursement were assessed through ordinary least squares regression. Statistical analysis was performed using Stata 18 (StataCorp).

## Results

Among 1673 OPPS 340B hospitals, 1325 (79%) received payment (median [range], $1.8 [4.0-17.0] million). Hospitals receiving payment had higher operating revenues (mean [SD], $2.34 [3.06] billion vs $1.22 [1.78] billion), were less likely to be rural (24.8% [329/1325] vs 56.6% [197/348]) or publicly owned (20.2% [268/1325] vs 28.7% [100/348]), and were more commonly teaching hospitals (56.2% [744/1325] vs 20.4% [71/348]) (Table). No difference was found in uncompensated care burden and operating margin by expected payment status. However, a 1–percentage point increase in repayments as a proportion of operating revenue was associated with a 2.1–percentage point decrease in uncompensated care burden and a 2.6–percentage point increase in operating margins (Figure). The nondrug share of total Part B reimbursements was negatively associated with repayment as a proportion of operating revenue.

**Table.** Characteristics and Safety-Net Focus of 340B Hospitals by Lump Sum Payment Size

Variable	No. (%), N = 1673^a^		Difference (payment vs no payment)	*P* value
	No payment (n = 348)	Payment (n = 1325)		
Net operating revenue, mean (SD), millions of $^b^	1215 (1785)	2343 (3059)	1128	<.001
Uncompensated care burden, mean (SD), %^c^	5.6 (5.1)	4.8 (6.4)	−0.7	.06
Total profit margin, mean (SD), %^d^	3.4 (10.0)	3.0 (12.8)	−0.4	.59
Rural, %^e^	197 (56.6)	329 (24.8)	−31.8	<.001
Public ownership, %^f^	100 (28.7)	268 (20.2)	−8.5	.001
Teaching status, %^g^	71 (20.4)	744 (56.2)	35.7	<.001

^a^ Includes 340B hospitals paid through the Medicare Outpatient Prospective Payment System participating in 340B for at least 1 year between 2018 and 2022, linked via Medicare certification number to Centers for Medicare & Medicaid Services Hospital Cost Report and the Addendum AAA of Outpatient Prospective Payment System proposed rule 1793-P.

^b^ Revenue derived from inpatient and outpatient care, net of any negotiated discounts.

^c^ Sum of charity care and bad debt costs divided by net operating revenue.

^d^ Total net revenue minus total expenses divided by total net revenue.

^e^ Nonmetropolitan county status based on Rural-Urban Continuum Codes.

^f^ Owned by state or local government.

^g^ Reporting indirect medical education payments.

**Figure.**
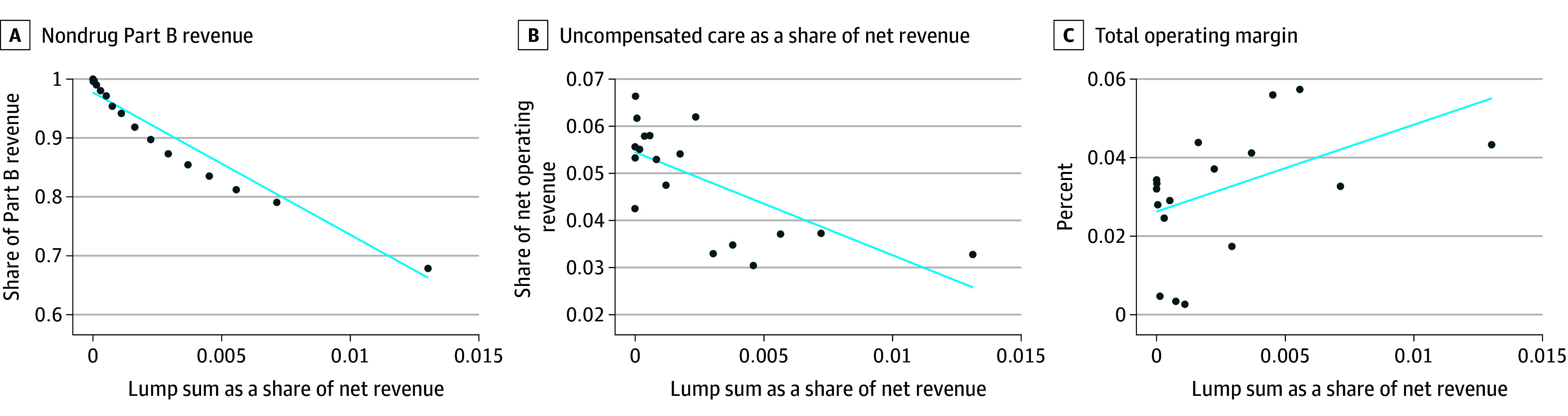
Associations Between Medicare Part B Reimbursement, Uncompensated Care, Total Margin, and Lump Sum Payments Associations among Medicare Part B reimbursement, uncompensated care, total margin, and lump sum payments were analyzed from a sample of 1673 hospitals in the 340B Drug Pricing Program (2018-2022). Lump sum payments were presented as a percentage of net operating revenue. Nondrug Part B reimbursement was calculated. The scatter plots show mean outcomes in lump sum amount bins. The estimated ordinary least squares regression coefficients for lump sum payments in each regression are as follows: for nondrug share of total Part B reimbursement (β = −23; *P* < .001), for uncompensated care burden (β = −2.1; *P* < .001), and for total margin (β = 2.6; *P* = .005).

## Discussion

One-fifth of OPPS 340B hospitals are not expected to receive a lump sum payment under CMS’s remedy for 340B-specific cuts to drug reimbursement. Disproportionately rural, publicly owned, and nonacademic hospitals will face cuts to nondrug Medicare Part B reimbursement without an offsetting lump sum repayment. Hospitals expected to receive smaller repayments receive proportionally more Medicare Part B reimbursement for nondrug services and are less financially stable than those with larger repayments. Study limitations included reliance on administrative data not subject to generally accepted accounting principles. Overall, these data suggest that the CMS repayment proposal may unintentionally harm vulnerable 340B hospitals while rewarding less vulnerable ones.

## Notes

Supplement. Data Sharing Statement
