# Supplementary material for: Characteristics of 340B Hospitals Receiving Medicare Part B Repayments
Source: JAMA Health Forum. 2024 Apr 26;5(4):e235397. doi: 10.1001/jamahealthforum.2023.5397 (PMC11065161; doi:10.1001/jamahealthforum.2023.5397)
Supplement: Supplement. — Data Sharing Statement [file jamahealthforum-e235397-s001.pdf]

Data Sharing Statement

Nikpay. Characteristics of 340B Hospitals Receiving Medicare Part B Repayments. *JAMA Health Forum*. Published April 26, 2024. doi:10.1001/jamahealthforum.2023.5397

Data
Data available: Yes
Data types: Data (not involving human participants), Other (please specify)
Additional Information: All data used for this project are publicly available.
How to access data: The datasets include Addendum AAA data: https://www.cms.gov/medicare/medicare-fee-service-payment/hospitaloutpatientpps/hospital-outpatient-regulations-and/cms-1793-p HCRIS data: https://www.cms.gov/data-research/statistics-trends-and-reports/cost-reports/cost-reports-fiscal-year 3400B Office of Pharmacy Affairs Database reports: https://340bopais.hrsa.gov/Reports Hospitals Participating in OPPS: https://data.cms.gov/provider-summary-by-type-of-service/medicare-outpatient-hospitals
When available: With publication

Supporting Documents
Document types: None

Additional Information
Who can access the data: Individuals seeking the data can download it freely from the sites I have listed above.
Types of analyses: See above - publicly available data
Mechanisms of data availability: See above - publicly available data
Any additional restrictions: See above - publicly available data
